# Burden of respiratory syncytial virus bronchiolitis on the Dutch pediatric intensive care units

**DOI:** 10.1007/s00431-021-04079-y

**Published:** 2021-04-23

**Authors:** Rosalie S. Linssen, Reinout A. Bem, Berber Kapitein, Katrien Oude Rengerink, Marieke H. Otten, Bibiche den Hollander, Louis Bont, Job B. M. van Woensel, on behalf of the PICE Study Group, Roelie M. Wösten-van Asperen, Richard H. Klein, Martin C. J. Kneyber, Jan Willem Kuiper, Carin Verlaat, Marc van Heerde, Maaike A. Riedijk, Dick A. van Waardenburg

**Affiliations:** 1 Pediatric Intensive Care Unit, Emma Children’s Hospital, Amsterdam University Medical Centers, location AMC, Meibergdreef 9, 1105 Amsterdam, AZ the Netherlands; 2 Department of Pediatric Infectious Diseases and Immunology, Wilhelmina Children’s Hospital, University Medical Center Utrecht, Utrecht, the Netherlands; 3 Department of Biostatistics and Research Support, Julius Center for Health Sciences and Primary Care, University Medical Center Utrecht, Utrecht, the Netherlands; 4 UMCU Laboratory of Translational Immunology, University Medical Center Utrecht, Wilhelmina Children’s Hospital, Utrecht, the Netherlands; 5 Respiratory Syncytial Virus Network (ReSViNET) Foundation, Zeist, the Netherlands

**Keywords:** Airway management, Child, Bronchiolitis, High flow nasal cannula, Non-invasive ventilation, Respiratory syncytial viruses, Vaccination

## Abstract

Respiratory syncytial virus (RSV) bronchiolitis causes substantial morbidity and mortality in young children, but insight into the burden of RSV bronchiolitis on pediatric intensive care units (PICUs) is limited. We aimed to determine the burden of RSV bronchiolitis on the PICUs in the Netherlands. Therefore, we identified all children ≤ 24 months of age with RSV bronchiolitis between 2003 and 2016 from a nationwide PICU registry. Subsequently we manually checked their patient records for correct diagnosis and collected patient characteristics, additional clinical data, respiratory support modes, and outcome. In total, 2161 children were admitted to the PICU for RSV bronchiolitis. The annual number of admissions increased significantly during the study period (β 4.05, SE 1.27, *p* = 0.01), and this increase was mostly driven by increased admissions in children up to 3 months old. Concomitantly, non-invasive respiratory support significantly increased (β 7.71, SE 0.92, *p* < 0.01), in particular the use of high flow nasal cannula (HFNC) (β 6.69, SE 0.96, *p* < 0.01), whereas the use of invasive ventilation remained stable.

*Conclusion*: The burden of severe RSV bronchiolitis on PICUs has increased in the Netherlands. Concomitantly, the use of non-invasive respiratory support, especially HFNC, has increased.

**Table:** 

**What is Known:** *• RSV bronchiolitis is a major cause of childhood morbidity and mortality and may require pediatric intensive care unit admission.* *• The field of pediatric critical care for severe bronchiolitis has changed due to increased non-invasive respiratory support options.*	
**What is New:** *• The burden of RSV bronchiolitis for the Dutch PICUs has increased. These data inform future strategic PICU resource planning and implementation of RSV preventive strategies.* *• There was a significant increase in the use of high flow nasal cannula at the PICU, but the use of invasive mechanical ventilation did not decrease.*

**Supplementary Information:**

The online version contains supplementary material available at 10.1007/s00431-021-04079-y.

## Introduction

With a worldwide hospitalization rate of over three million children and an in-hospital mortality of up to 75,000 cases annually, respiratory syncytial virus (RSV) bronchiolitis is a leading cause of childhood morbidity and mortality [1, 2]. Although hospitalization numbers for RSV bronchiolitis seem to be decreasing, bronchiolitis-associated healthcare costs have increased in several countries since 2000 [3–5]. A possible reason for these rising costs may be increased usage of pediatric intensive care unit (PICU) facilities [3, 4, 6]. Although only 2% of children with RSV bronchiolitis require PICU admission for mechanical ventilation, it is estimated that PICU care accounts for 18% of the total RSV-related hospital costs [3]. The number of PICU admissions may be influenced by changing admission thresholds [4, 7], in addition to an increasing variety of available respiratory support modalities, but only limited insight into these trends over time is available.

RSV prevention strategies with (extended) monoclonal antibodies focus on children at risk for severe RSV bronchiolitis, such as those born prematurely or with comorbidity [8–10]. Yet, most children with RSV bronchiolitis admitted to a PICU may not have a qualifying risk factor [11, 12].

In order to provide further insight into the burden of RSV bronchiolitis on the PICU, we aimed to study the burden of severe RSV bronchiolitis in the Netherlands by exploring the characteristics and PICU course of children admitted to all Dutch PICUs over a 13-year period.

## Methods

We defined the burden of RSV bronchiolitis primarily as the number of PICU admissions for RSV bronchiolitis and used the need for respiratory support and information on PICU resource use, morbidity and mortality as secondary parameters to describe this burden. Children with RSV bronchiolitis were identified through the multicenter national PICU registry (the Dutch pediatric intensive care evaluation, PICE) which covers the full national PICU caseload. Pediatric critical care in the Netherlands is exclusively provided in university medical centers. Children with bronchiolitis are referred to a PICU when they develop signs of imminent respiratory insufficiency or central apneas with insufficient oxygenation and/or ventilation, despite oxygen supply via low flow or high flow nasal cannula (HFNC). HFNC was introduced in the PICUs and general hospitals in the Netherlands from 2009 to 2010 onwards. At introduction, there were no clear guidelines on the use of HFNC. To interpret data on PICU admissions for RSV bronchiolitis within trends in overall PICU admissions in the Netherlands, we additionally extracted data on the number of all PICU admissions in children aged ≤ 24 months and ≤ 18 years from the PICE registry. Detailed information on the eight PICUs in the Netherlands is provided in the Supplemental eMethods. The study was reviewed by the Utrecht University Medical Center Institutional Review Board (number 17-851/C) and deemed exempt.

### Case identification and data extraction

We identified all patients with a primary, or an associated, code of “bronchiolitis” or “respiratory syncytial virus” from the PICE registry (Supplemental eMethods). Subsequently, medical records of all identified patients were checked manually for correct diagnosis and details of clinical course. Since we aimed to study RSV bronchiolitis-related PICU burden, we only included patients with a laboratory-confirmed RSV infection (as detected by a rapid antigen test or PCR) and who presented with the clinical symptoms of bronchiolitis according to the American Academy of Pediatrics and/or central apneas [13]. Patients with a co-infection with another respiratory virus were included, as were patients with pre-existing tracheostomy and who received respiratory support at home prior to PICU admission. In those patients, the end of respiratory support was defined as the moment when they received respiratory support in the same range as prior to PICU admission. Duplicates, e.g., due to transfers between PICUs and re-admissions for the same disease episode, were excluded. We collected data on demographics, medical history, microbiology, respiratory support modes (including the last mode in the referring hospital before PICU admission), and clinical course. To further study patient dynamics for the different age groups in our cohort, we identified three subgroups: children 0–3 months, 4–12 months, and 13–24 months old on PICU admission.

Comorbidity was defined as the presence of a medical condition according to the ICD-10-CM codes in the following categories: neuromuscular, cardiovascular, respiratory, renal, gastrointestinal, hematology or immunologic, metabolic, malignancy, and congenital or genetic disorders. Prematurity was defined as birth before 37 weeks of gestational age.

We made the distinction between invasive mechanical ventilation (IMV) via an endotracheal tube and non-invasive modes of respiratory support that included HFNC, nasal continuous positive airway pressure (nCPAP, with a nasal mask as interface), and non-invasive mechanical ventilation (NIV, with a facemask as interface). NIV was defined as either inspiratory pressure support upon spontaneous breathing or intermittent mandatory pressure- or volume-controlled ventilation.

### RSV surveillance and data on national population at risk

To compare the number of PICU admissions with the incidence of RSV among the general population, we collected national surveillance data on viral respiratory infections (data provided by the working group for Clinical Virology of the Dutch Society for Medical Microbiology, Supplemental eMethods). In order to calculate population-based estimates per 100,000 children for (1) RSV bronchiolitis PICU admissions in children ≤ 24 months old, (2) all PICU admissions in children ≤ 24 months old, and (3) children ≤ 18 years old, we collected the total number of children < 24 months of age and < 18 years of age living in the Netherlands from the Statistics Bureau of the Netherlands (CBS, Supplemental eMethods).

### Statistical analysis

Data are presented as numbers (n) and percentages (%), means, standard deviations (SD), or medians and interquartile ranges (IQR) where appropriate. We identified children who (1) developed RSV bronchiolitis during hospital or PICU stay for another reason (e.g., surgical patients) and (2) children with RSV bronchiolitis who had a prolonged length of stay due to another reason (e.g., underlying neuromuscular disorder, cardiac surgery during the PICU stay). For PICU length of stay and IMV analyses, we determined the IQR in days, and values were capped off at a fixed value of 25 days to avoid overestimation.

We performed linear regression modelling on time trends for number of admissions, children with a comorbidity, and modes of respiratory support. As RSV infections occur predominantly in the winter, data on RSV admissions and national surveillance data were shifted into “seasons” from July 1 up to and including June 30 the year thereafter. For these time trend analyses, we excluded data from January up to July 2003 and from July up to January 2017, as they represent a half “RSV” season. We performed a subanalysis for the period before and after the introduction of HFNC (2009) for the use IMV. Due to Dutch privacy regulations, we could not check for repeated admissions in the same patient, when the second admission took place in a different PICU in a consecutive year. However, we expected this number to be negligible, and therefore, we did not account for potential dependence in the model. Betas represent the change in the outcome variable (PICU admission) for every 1-unit change in the predictor variable (year) and are provided with standard errors (SE). Chi-square tests were used to compare between categorical data (including differences in patient characteristics and use of IMV between PICUs and to compare the use of different modes of respiratory support before and after the introduction of HFNC). Where appropriate, Kruskal-Wallis tests are reported for continuous data. Statistical analysis was performed using SPSS 26; significance was concluded when *p* < 0.05.

## Results

**Fig. 1:**
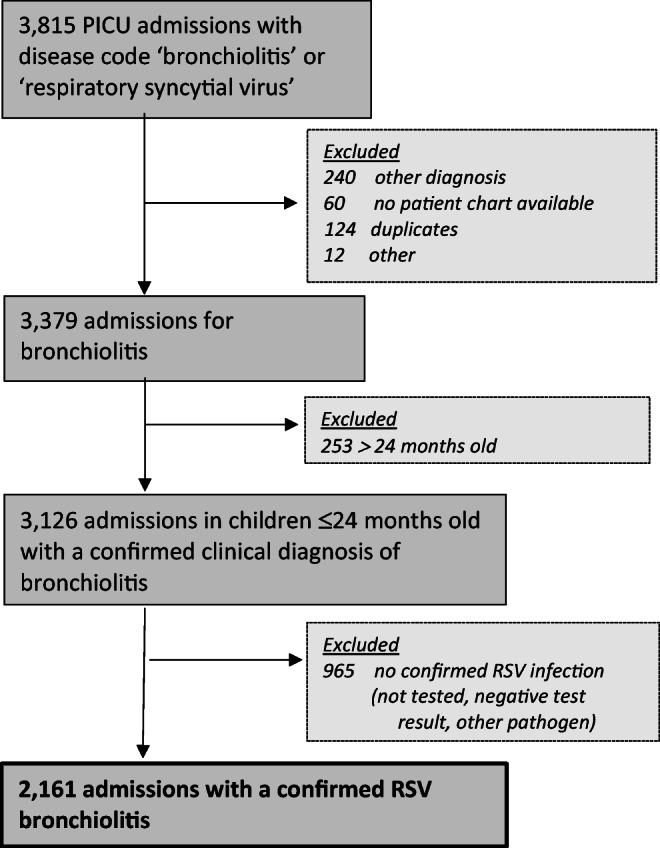
Flowchart of eligible, excluded, and finally included patients identified in the national PICU database (PICE)

From a total of 3815 records from the PICE registry 2003–2016 (Supplemental eTable 1), we identified 2161 (69.1%) children ≤ 24 months of age with confirmed RSV bronchiolitis. Reasons for exclusion are given in Fig. 1. Patient characteristics are presented in Supplemental eTable 2. A subgroup of 32 (1.5%) children suffered from nosocomial RSV bronchiolitis (median length of stay 13.5 days, IQR 8.5–24.3). There were some significant differences in patient characteristics and frequency of IMV between the eight PICUs (eMethods 1 and Supplemental eFig. 1).

**Fig. 2:**
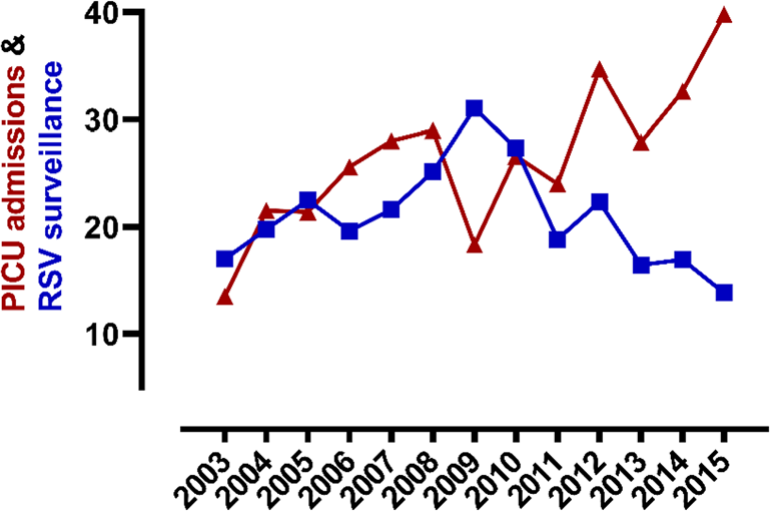
Annual number of PICU admissions for confirmed RSV bronchiolitis per 100,000 children aged < 24 months among the Dutch population and annual national RSV surveillance data × 1000. Red line: PICU admissions for RSV bronchiolitis per 100,000 children; blue line: surveillance data on RSV isolations among the Dutch population. X-axis: “2003” refers to the RSV season 2003–2004, “2004” refers to the RSV season 2004–2005, etc. (surveillance data on all respiratory viruses is presented in eFig. 3)

**Fig. 3:**
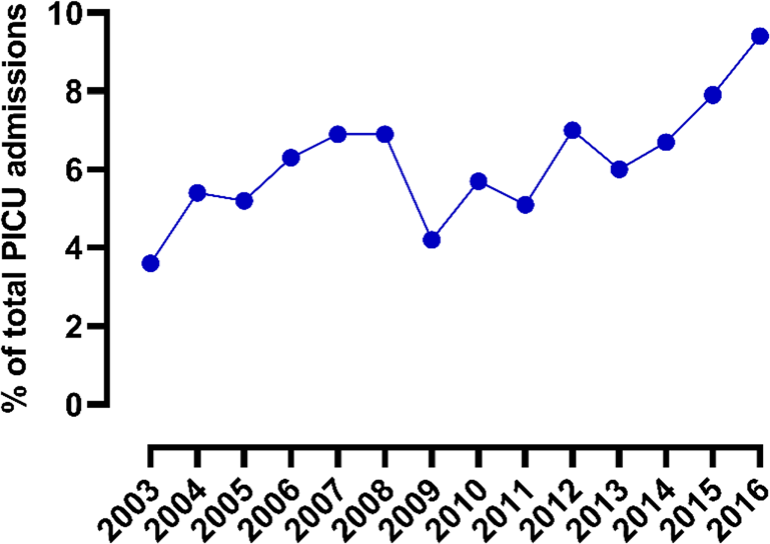
Annual number of PICU admissions for RSV bronchiolitis of children ≤ 24 months old as a % of total number of PICU admissions of children ≤ 24 months old in the Netherlands

The number of RSV bronchiolitis PICU admissions increased significantly (β 4.05, SE 1.27, *p* = 0.01) during the study period (Table 1, Figs. 2 and 3). Over the same period, the number of RSV isolations among the population did not change (β − 22.77, SE 33.49, *p* = 0.51, *n* = 27,227 isolates, Fig. 2), whereas the number of children aged < 24 months among the Dutch population decreased from 608,896 on January 1, 2004, to 518,457 on January 1, 2016 (β − 6232.12, SE 535.45, *p* < 0.01). Subsequent population-based estimates on the number of PICU admissions for RSV bronchiolitis showed a fourfold increase, from 13.5 per 100,000 children in 2003 to 48.0 per 100,000 children in 2016 (Table 1, Fig. 2). In total, 1293 (59.8%) children had no comorbidity and were born term, whereas 868 (40.2%) children had a comorbidity and/or were born prematurely. Neither the number of children admitted to the PICU with a comorbidity nor those born prematurely changed significantly over time (β − 0.05, SE 0.44, *p* = 0.91 and β − 0.23, SE 0.47, *p* = 0.81 respectively, Supplemental eFig. 2). More detailed information on comorbidities is presented in Supplemental eTable 2.

**Table 1:** Population-based estimates of PICU admissions for RSV bronchiolitis in the Netherlands per 100,000 children per year from 2003 to 2016

Year	Number of PICU admissions (n)			PICU admissions per 100.000 children		
	RSV bronchiolitis in children ≤ 24 months	All children ≤ 24 months N (%)	All children ≤ 18 years N (%)	RSV bronchiolitis < 24 months	All children < 24 months	All children < 18 years
*2003*	83	2279 (3.6%)	4273 (1.9%)	13.5	369.7	113.0
*2004*	131	2391 (5.4%)	4562 (2.9%)	21.5	392.7	120.2
*2005*	128	2449 (5.2%)	4727 (2.7%)	21.4	409.6	124.6
*2006*	149	2351 (6.3%)	4723 (3.2%)	25.6	403.9	125.0
*2007*	158	2275 (6.9%)	4861 (3.3%)	28.0	402.5	129.3
*2008*	160	2312 (6.9%)	4821 (3.3%)	29.0	418.4	128.8
*2009*	101	2408 (4.2%)	5202 (1.9%)	18.3	437.0	139.3
*2010*	147	2537 (5.7%)	5514 (2.7%)	26.6	459.7	148.2
*2011*	133	2617 (5.1%)	5730 (2.3%)	24.0	471.5	154.6
*2012*	191	2729 (7.0%)	6041 (3.2%)	34.7	496.1	163.8
*2013*	151	2529 (6.0%)	5762 (2.6%)	27.9	467.9	157.2
*2014*	172	2572 6.7%)	5762 (3.0%)	32.6	487.9	158.3
*2015*	208	2643 (7.9%)	5719 (3.6%)	39.8	505.7	157.7
*2016*	249	2662 (9.4%)	5750 (4.3%)	48.0	513.4	159.0

### Identification of different age groups in children admitted to the PICU for RSV bronchiolitis

We identified 1697 (78.5%) children aged 0–3 months, 359 (16.5%) children aged 4–12 months, and 105 (4.9%) children aged 13–24 months on PICU admission. Of the children who were 0–3 months old on PICU admission, 1152 children were also born term (53.3% of total PICU bronchiolitis admissions). The proportion of children 0–3 months old on PICU admission increased significantly over time (β 4.39, SE 1.65, *p* = 0.02), as well as the proportion of children 4–12 months old (β 1.17, SE 0.38, *p* < 0.01), while the proportion of children 13–24 months old did not change (β 0.31, SE 0.22, *p* = 0.18).

### Temporal dynamics of respiratory support

**Fig. 4:**
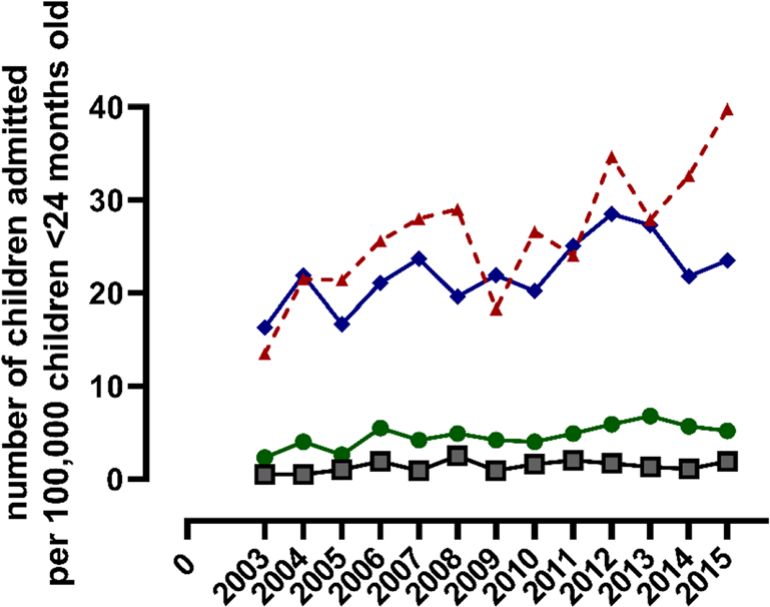
Age groups among the children admitted to the PICU for RSV bronchiolitis. The number of children in the different age groups are displayed per 100,000 children aged < 24 months among the Dutch population. Red line: PICU admissions for RSV bronchiolitis per 100,000 children; blue line: children aged 0–3 months old, green line: children aged 4–12 months old, grey line: children aged 13–24 months old. X-axis: “2003” refers to the RSV season 2003–2004, “2004” refers to the RSV season 2004–2005, etc.

In 1551 (71.8%) children, IMV was applied, which was the first mode of respiratory support in 1449 of them (Table 2). The number of children requiring IMV did not change significantly over time (β 0.90, SE 0.32, *p* = 0.48, Fig. 4). Non-invasive respiratory support (i.e., HFNC, nCPAP, or NIV) was applied in 631 (29.2%) children and was the primary mode of support in 409 children (Table 2). The overall use of non-invasive respiratory support at the PICU as the primary mode increased significantly over time for all modes (β 7.71, SE 0.92, *p* < 0.01, Fig. 4); in particular the use of HFNC increased (β 6.69, SE 0.96, *p* < 0.01). The number of children who received multiple modes of respiratory support was higher from 2010–2016 compared to 2003–2009 (*p* < 0.01). The slopes for IMV in the subanalysis on time trends before and after the introduction of HFNC did not change significantly (Table 3).

**Table 2:** Applied modes of respiratory support as the initial form of support and at any time point during admission

	HFNC		nCPAP		NIV		IMV	
	*Initial*	*Any timepoint*	*Initial*	*Any timepoint*	*Initial*	*Any timepoint*	*Initial*	*Any timepoint*
All children (*n* = 2161)	278 (12.9%)	395 (18.3%)	75 (3.5%)	132 (6.1%)	56 (2.6%)	104 (4.8%)	1449 (67.1%)	1551 (71.8%)

*HFNC* high flow nasal canula, *nCPAP* nasal continuous positive airway pressure, *NIV* non-invasive mechanical ventilation, *IMV* invasive mechanical ventilation

Use of low flow oxygen supply was not scored. Multiple modes of support may have been given. The sum of treatments does not represent the number of children receiving > 1 mode of respiratory support

**Table 3:** Subanalysis on the trends in use of IMV before and after the introduction of HFNC in 2009–2010

	Before HFNC introduction 2003–2009	After HFNC introduction 2010–2016
IMV as the initial mode of support	β 4.29, SE 3.73, p =0.31	β -0.79, SE 3.38, p =0.83
IMV during PICU stay (any timepoint)	β 4.57, SE 3.62, p =0.28	β 1.61, SE 0.21, p =0.66

Legend: *HFNC* high flow nasal canula, *IMV* invasive mechanical ventilation

**Fig. 5:**
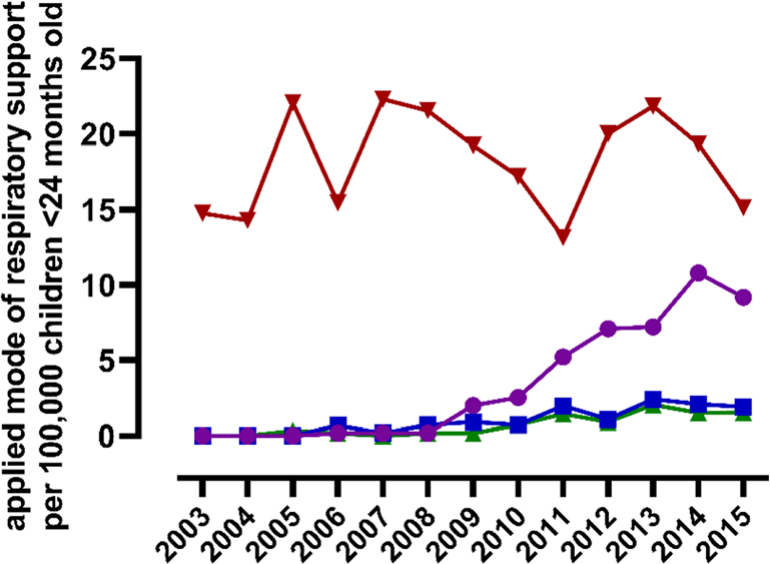
Initially applied modes of respiratory support for patients admitted to a Dutch PICU for RSV bronchiolitis per 100,000 children aged < 24 months old. Red line: invasive mechanical ventilation; purple line: high flow nasal cannula; blue line: nasal continuous positive airway pressure; green line: non-invasive ventilation. X-axis: “2003” refers to the RSV season 2003–2004, “2004” refers to the RSV season 2004–2005, etc.

Information on respiratory support before PICU referral was available in 307 (13.9%) children. Of these, 221 received HFNC and 86 nCPAP, and the application of both modalities before PICU admission increased significantly over time (HFNC: β 5.60, SE 1.1, *p* < 0.01 and CPAP: β 0.66, SE 0.2, *p* = 0.02) (Fig. 5).

### Outcome

Median length of PICU stay was 8 (IQR 5–11) days, which decreased slightly from 2003 to 2016 (β − 0.10 SE 1.33 *p* = 0.02). The median duration of IMV among children who received this as the initial mode of support was 7 (IQR 5–10) days. Of the 1551 children who had received IMV, 192 (8.9%) were on high-frequency oscillatory (HFO) ventilation. In total, 236 (15.2%) needed to be re-intubated, and reasons for re-intubation included post-intubation upper airway obstruction (*n* = 79, 33.5%), pulmonary deterioration (*n* = 32, 13.6%), accidental extubation (*n* = 19, 8.1%), and miscellaneous (e.g., withdrawal of sedation, unexpected bronchospasms, cardiovascular or other complications, *n* = 106, 44.9%). Non-reversible post-extubation upper airway obstruction (such as stenosis) was reported in 30 (1.9%) children, in whom a tracheostomy was indicated in eight, while a tracheostomy was performed in another nine children for other reasons. ECMO was applied in 32 (1.5%) children. In total, 37 (1.7%) children died, of whom 27 (73.0%) had at least one comorbidity, and 11 (29.7%) were born prematurely. Mortality did not change over time. Comparisons for the different outcomes and resource use between the different age groups are shown in the Supplemental eTable 3.

## Discussion

This study determined the nationwide burden of severe RSV bronchiolitis on all eight PICUs in the Netherlands between 2003 and 2016. We observed a fourfold increase in PICU admissions for RSV bronchiolitis per 100,000 children ≤ 24 months of age, despite a stable number of RSV isolations among the general population.

The increase in PICU admissions reported in this study is higher than the twofold increase previously reported for specialized PICUs in Australia [4] and adds to the presumptive evidence that PICU burden of RSV bronchiolitis in developed countries has increased over the last decade [3–6]. The observed increase in PICU admissions was unlikely driven by increased comorbidity, as the comorbidity rate remained stable over time and is comparable with the rate reported in previous literature [4, 6, 14]. Interestingly, an observational study reporting on seven European countries did not report an increase in general ward hospital admission rates for RSV bronchiolitis [15].

The observed rise in PICU admissions for bronchiolitis in our study was accompanied by an increase in the number of children who received non-invasive respiratory support, while the number of patients receiving IMV remained stable. The management of severe RSV bronchiolitis in the PICU relies mainly on supportive care. Over the last decades, respiratory support options have evolved in terms of nCPAP and bi-level non-invasive respiratory support modes using different supply interfaces, [7] but the introduction of HFNC has been considered the most popular advancement [7]. The increased use of non-invasive respiratory support, and especially HFNC, was previously described in studies from Australia and the USA [4, 5]. Although some of those studies also reported a decreased use of IMV [4, 16], this was, similar to a study from the USA [5], not the case in our study. Literature on HFNC focuses on the use of HFNC in general pediatric wards, showing improvement in vital signs in children with RSV bronchiolitis [17, 18]. In these studies, a beneficial role for the use of HFNC in the general pediatric ward to prevent of PICU admissions is suggested (19, 20), but the effectiveness of preventing PICU admission and intubation rates is under discussion [19, 20]. A recent survey in the UK indicated however that treating pediatricians prefer HFNC over CPAP as being more effective with fewer complications [21]

In our study, we did not find a decrease in PICU admissions for RSV bronchiolitis after the introduction of HFNC in 2009, precluding any conclusions on the effectiveness of HFNC in the prevention of escalation of respiratory support [19, 20]. Although current study was not designed to investigate the effectiveness of HFNC, our observation of the increased burden of RSV-bronchiolitis for the PICU concomitantly with the wide-scale introduction of HFNC warrants future research on this topic. The increase in non-invasive respiratory support options to treat these children at the PICU may have influenced PICU admission thresholds or referral policy, but without larger-scale epidemiological intervention studies, we can only speculate.

The intubation rate in our study is higher than in previous studies [4, 22–24], while our re-intubation rate is comparable to previously reported re-intubation rates [25, 26]. A large-scale observational study in Australia reported a 37% intubation rate in children admitted to the PICU in 2002 [4]. However, this study included children admitted to both general ICUs as well as specialized PICUs, and Australian demographics and infrastructure are not comparable with those of the Netherlands. Intubation rates reported by the different PICUs in our study varied and reasons for the higher intubation rates remain speculative.

### Possible impact of a vaccination strategy on RSV PICU burden

The increased number of PICU admissions for RSV bronchiolitis was mostly driven by increased admissions in children up to 3 months old. Although no significant differences between the age groups studied in our cohort were found in terms of mortality, the high proportion of very young children admitted to the PICU follows previous literature that the risk for a severe course of RSV bronchiolitis is highest in the first months of life [1]. Interestingly, we observed that over half of the children admitted to the PICU for RSV bronchiolitis was ≤ 3 months old at the moment of admission and born term. The combination of the observed increase in PICU admissions that was mostly driven by the very young, in combination with high intubation rates in this group compared to older children and the overall PICU-related morbidity reported in this study, underlines the need for a more comprehensive prevention strategy. Such prevention strategy should also include children in their first months of life and should not only include children with risk factors for severe RSV disease such as premature birth. This is important in the light of recent developments in the field of vaccination research: a randomized trial on extended half-life antibodies showed fewer hospitalizations for RSV infections compared to placebo but was only carried out in preterm infants [10]. A recently published maternal vaccination trial carried out in term babies on RSV-fusion protein nanoparticles showed 44% efficacy in preventing RSV-hospitalizations, but did not meet its primary outcome: preventing medically significant lower respiratory tract infection during the first 90 days of life [27, 28]. As over 90% of RSV related mortality occurs in the less developed countries [1], most studies focus on these countries while estimating the possible impact of a vaccination strategy. Current study adds to these impact estimations, as our findings implicate that a major impact may be expected from a preventive strategy that protects the very young and those without risk factors for countries with access to a PICU as well. Moreover, as the prevention of (severe) RSV infection will also result in lower general hospital admission rates for RSV bronchiolitis, this will most likely decrease the pressure on healthcare resources on an even larger scale.

### Strengths and limitations

A major strength of this study was that, compared to registry studies reporting on RSV bronchiolitis, all diagnoses, serology, and clinical outcomes were confirmed by manual checking of the individual patient records. It presents nationwide data collected from a large patient cohort collected over a 13-year timeframe. Yet, we need to address several limitations. First, we identified only patients who were coded under the diagnosis “bronchiolitis” or “respiratory syncytial virus.” Although the accuracy of the PICE database is checked regularly by tracers, incorrect input in the PICE database may have led to missed patients. Viral testing is not routinely performed in all admitting hospitals in the Netherlands, and information in the patient charts might have been incomplete. This may have led to underestimation of RSV-attributable cases. Second, no information on the total group of children hospitalized with RSV bronchiolitis was available, and as such, assumptions on the cause of the increased PICU burden remain speculative. Last, regional differences may exist, and these may have been influenced by the infrastructural differences between PICUs.

## Conclusion

The PICU disease burden of RSV bronchiolitis in the Netherlands increased between 2003 and 2016, concomitantly with the introduction and availability of non-invasive respiratory support modalities. A vaccination strategy addressing both term and preterm born children up to 3 months of age may have a substantial impact on the burden of RSV bronchiolitis on PICUs.

## Supplementary Information

ESM 1 (DOCX 237 kb)

## Data Availability

Data may be shared upon reasonable requests

## Abbreviations

ANZPIC: Australian and New Zealand Pediatric Intensive Care
CPAP: Continuous positive airway pressure
ECMO: Extracorporeal membrane oxygenation
HFNC: High flow nasal cannula
IMV: Invasive mechanical ventilation
NIV: Non-invasive mechanical ventilation
PICU: Pediatric intensive care unit
RSV: Respiratory syncytial virus
