# Supplementary material for: Burden of respiratory syncytial virus bronchiolitis on the Dutch pediatric intensive care units
Source: Eur J Pediatr. 2021 Apr 23;180(10):3141–9. doi: 10.1007/s00431-021-04079-y (PMC8429147; doi:10.1007/s00431-021-04079-y)
Supplement: Supplementary file 1 — (DOCX 237 kb) [file 431_2021_4079_MOESM1_ESM.docx]

**Supplemental Digital Content**

**Full title:** Burden of Respiratory Syncytial Virus bronchiolitis on the Dutch pediatric intensive care units

**Journal**: European Journal of Pediatrics

**Authors***:* Rosalie S. Linssen MD ^1^, Reinout A. Bem MD PhD ^1^, Berber Kapitein MD PhD ^1^, Katrien Oude Rengerink PhD^2,3^, Marieke H. Otten MD PhD ^1^, Bibiche den Hollander BSc^1^, Louis Bont MD PhD ^2,4,5^, Job B.M. van Woensel MD PhD ^1^, on behalf of the PICE Study Group*

**Collaborators:** the Pediatric Intensive Care Evaluation (PICE) study group: Roelie M. Wösten-van Asperen, Richard H. Klein, Martin C.J. Kneyber, Jan Willem Kuiper, Carin Verlaat, Marc van Heerde, Maaike A. Riedijk, Dick A. van Waardenburg.

**Author Affiliations**

1. Pediatric Intensive Care Unit, Emma Children’s Hospital, Amsterdam University Medical Centers, location AMC, the Netherlands
2. Department of Pediatric Infectious Diseases and Immunology, Wilhelmina Children’s Hospital, University Medical Center Utrecht, the Netherlands
3. Department of Biostatistics and Research Support, Julius Center for Health Sciences and Primary Care, University Medical Center Utrecht, Utrecht, The Netherlands
4. UMCU Laboratory of Translational Immunology, University Medical Center Utrecht, the Netherlands; Department of Pediatrics, Wilhelmina Children's Hospital, University Medical Center Utrecht, the Netherlands.
5. Medical Research Council: Respiratory and Meningeal Pathogens Research Unit and Department of Science and Technology/National Research Foundation: Vaccine Preventable Diseases, University of the Witwatersrand, Johannesburg, South Africa; Respiratory Syncytial Virus Network (ReSViNET) Foundation, Zeist, the Netherlands.

*Corresponding author:* Ms. R.S. Linssen
[r.s.linssen@amsterdamumc.nl](mailto:r.s.linssen@amsterdamumc.nl)

**Supplemental Digital Content**

**Supplemental eMethods**

**eFigure 1** – Comparisons of the different PICUs

**Legend:** Panel 1 a-e: bar graphs with the number of patients presented as a percentage of the total number of patients admitted to that specific PICU. Each bar represents a different PICU.

Please note differences in y-axis ranges between graphs. Chi Square tests are used for differences in proportions between PICUs.

**eFigure 2 -** Number of children admitted to the PICU aged <24 months old with a comorbidity and with a medical history of premature birth per 100,000 children among the Dutch population from 2003-2016

**Legend**: blue line: children with a comorbidity; green line: children with a medical history of premature birth; red line: children with both a comorbidity and a medical history of premature birth.. X-axis: ‘2003’ refers to the RSV season 2003-2004, ‘2004’ refers to the RSV season 2004-2005 etc.

**eFigure 3 -** Surveillance data of RSV, Influenza A, B and C, Bocavirus and Human Metapneumovirus in the Netherlands during the study period

**Legend**: blue line: Influenza type C virus, red line: Influenza type B. Green line: Influenza type A virus. Purple line: Human Bocavirus *. Orange line: Respiratory Syncytial virus. Black line: Human Metapneumovirus. *surveillance data on Bocavirus were not collected before 2011.

X-axis: ‘2003’ refers to the RSV season 2003-2004, ‘2004’ refers to the RSV season 2004-2005 etc.

**eTable 1** – Overview of all extracted records and ANZPIC codes from the PICE registry 2003-2016

**eTable 2 -** Patient characteristics and comorbidity in 2,161 children ≤ 24 months of age with confirmed RSV bronchiolitis

**Legend:** ^a^ Due to the retrospective nature of the study, information on certain cardiac findings was sometimes not complete or difficult to interpret. As such, we additionally collected information of the cases in which the cardiac anomaly was substantial, or when it was documented as hemodynamic significant.

**eTable 3 -** Comparison between different age groups in children admitted to a Dutch PICU for RSV Bronchiolitis 2003-2016

**Legend**: Pearson Chi Square Tests were carried out to compare groups.. ^1^ Kruskall – Wallis Test instead of Pearson Chi Square Test. % are calculated using the number of children in the specific age group as the denominator. *Resuscitation refers to: cardiac and/or respiratory arrest with subsequent return of spontaneous breathing and/or circulation after medical intervention. NS: not significant.

**Supplemental eMethods**

*Additional information on the PICU landscape and differences between the eight PICUs in the Netherlands.*

There were eight PICUs in the Netherlands between 2003-2016 in the following University Medical Centers: Beatrix Children’s Hospital at the University Medical Center Groningen, Leiden Medical Center, Wilhelmina Children’s Hospital at the University Medical Center Utrecht, Radboud University Medical Center, Sophia Children’s Hospital at the Erasmus Medical Center, Rotterdam, Maastricht University Medical Center, Emma Children’s Hospital at the Academic Medical Center Amsterdam and VU Medical Center, Amsterdam. These PICUs provide level one patient care and comply with the quality of care recommendations of the Dutch Government [1]. Facilities and local regulations may differ between the PICUs. High-level surgical cardiac care is centralized in four PICUs. HFNC was introduced around 2009, but timing and availability may have differed between PICUs. This is also the case for the use of non-invasive ventilation. High-Frequency Oscillatory Ventilation was only regularly provided at two PICUs. ECMO is also only provided in two PICUs. When ECMO is deemed necessary, patients are referred to an ECMO providing PICU. Distances in the Netherlands are relatively small (the total surface area of the Netherlands is approximately 41,543 km^2^) and the choice of PICU at moment of patient (PICU) admission is based on medical needs, location of parents and availability of hospital beds during the winter months. All Dutch PICUs collaborate in the national PICE registry, the Pediatric Intensive Care Evaluation.

*Additional information on data extraction from the PICE registry*

The PICE registry can be found online at: <https://www.pice.nl>. The PICE database uses the Australian and New Zealand Pediatric Intensive Care (ANZPIC) registry uniform diagnostic codes to register the reasons for PICU admissions [2]. The ANZPIC registry also allows for registration of five associated diagnoses relevant for each PICU admission. In this way, we could also identify patients who had not primarily been registered under the diagnosis RSV bronchiolitis. We extracted all patients with a primary, or any associated, code of 'bronchiolitis' or ‘respiratory syncytial virus’ according to the ANZPIC definitions. In addition, we retrieved cases which had a diagnosis item of ‘bronchiolitis’ in the PIM 2 score.

*Additional information on the RSV surveillance data and national population data*

These viral surveillance data are collected through both hospital and regional reference laboratories spread across the country, which cover 29-44% of the Dutch population and report on all confirmed tests of 31 viruses [3]. An inventory study on RSV data indicated that these figures are mainly derived from children <6 months of age [3, 4].

Data on the total number of children <24 months of age living in the Netherlands (national population data) are published by the CBS on January 1^st^ each year. These data are publicly available from [www.cbs.nl](http://www.cbs.nl). CBS only provides data on children <24 months old, but we assumed these numbers to be very close to the number of children ≤24 months old.

**References**

1. Rosenberg DI, Moss MM. Guidelines and Levels of Care for Pediatric Intensive Care Units. *Pediatrics*. 2004;114(4):1114-1125.

2. Slater A, Shann F, McEniery J. The ANZPIC registry diagnostic codes: a system for coding reasons for admitting children to intensive care. *Intensive Care Med*. 2003;29(2):271-277.

3. Vos LM, Teirlinck AC, Lozano JE, et al. Use of the moving epidemic method (MEM) to assess national surveillance data for respiratory syncytial virus (RSV) in the Netherlands, 2005 to 2017 *Euro Surveill*. 2019;24(20).

4. van den Brandhof WE, Kroes ACM, Bosman A, M.F. P, Heijnen MLA. Rapportage van virologische diagnostiek in Nederland; representativiteit van de gegevens uit de virologische weekstaten*. Infectieziekten Bulletin*: Rijksinstituut voor Volksgezondheid en Milieu (RIVM);2002.

**eFigure 1** – Comparisons of the different PICUs


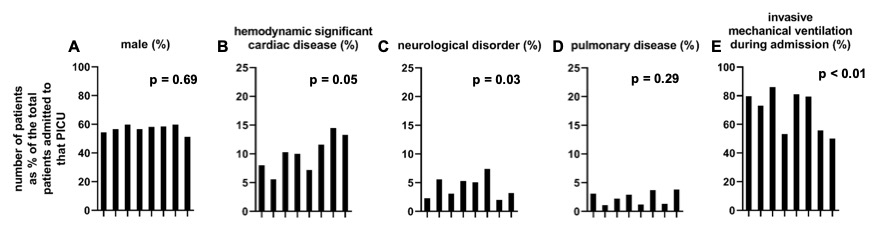


**Legend:** Panel 1 a-e: bar graphs with the number of patients presented as a percentage of the total number of patients admitted to that specific PICU. Each bar represents a different PICU.

Please note differences in y-axis ranges between graphs. Chi Square tests are used for differences in proportions between PICUs.

**eFigure 2 -** Number of children admitted to the PICU aged <24 months old with a comorbidity and with a medical history of premature birth per 100,000 children among the Dutch population from 2003-2016


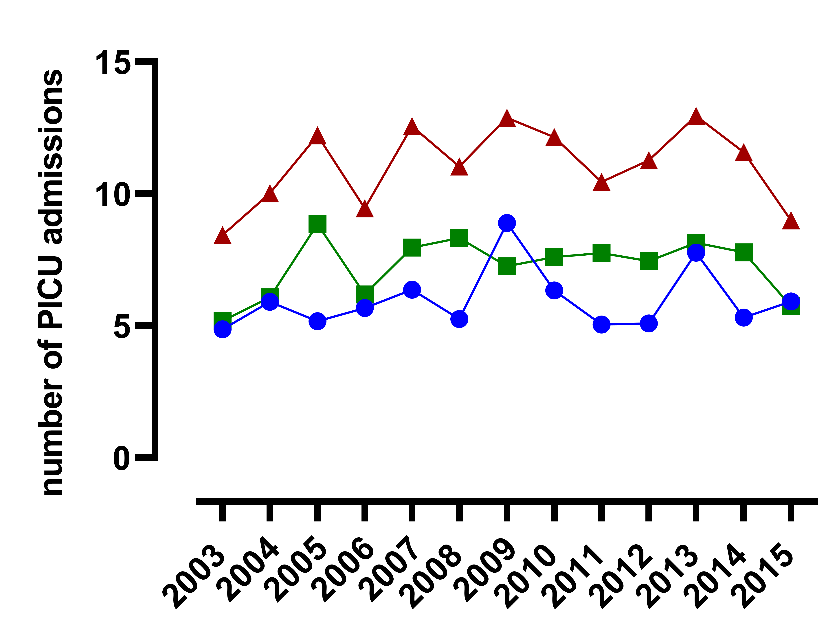


**Legend**: blue line: children with a comorbidity; green line: children with a medical history of premature birth; red line: children with both a comorbidity and a medical history of premature birth. X-axis: ‘2003’ refers to the RSV season 2003-2004, ‘2004’ refers to the RSV season 2004-2005 etc.

**eFigure 3 -** Surveillance data of RSV, Influenza A, B and C, Bocavirus and Human Metapneumovirus in the Netherlands during the study period

**
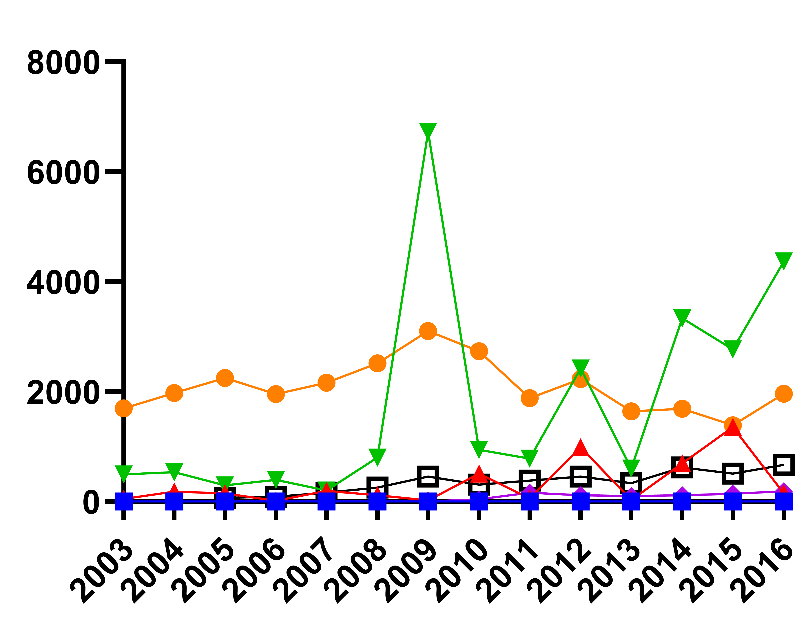
**

**Legend**: blue line: Influenza type C virus, red line: Influenza type B. Green line: Influenza type A virus. Purple line: Human Bocavirus *. Orange line: Respiratory Syncytial virus. Black line: Human Metapneumovirus. *surveillance data on Bocavirus were not collected before 2011.

X-axis: ‘2003’ refers to the RSV season 2003-2004, ‘2004’ refers to the RSV season 2004-2005 etc.

**eTable 1** – Overview of all extracted records and ANZPIC codes from the PICE registry 2003-2016

| **Main combination** | **Combination per sub code** | **Frequency** |
| --- | --- | --- |
| Bronchiolitis and RSV | Bronchiolitis (principal diagnosis) and RSV | 1,257 |
|  | Bronchiolitis (associated diagnosis) and RSV | 329 |
|  | Bronchiolitis PIM2 and RSV | 50 |
| Bronchiolitis without RSV | Bronchiolitis (principal diagnosis) without RSV | 1,318 |
|  | Bronchiolitis (associated diagnosis) without RSV | 280 |
|  | Bronchiolitis (PIM 2) without RSV | 199 |
| RSV without Bronchiolitis |  | 247 |
| Surgical or post-operative PICU admission and Bronchiolitis | Bronchiolitis (principal diagnosis) and surgical or postoperative PICU admission | 21 |
|  | Bronchiolitis (associated diagnosis) and surgical or post-operative PICU admission | 44 |
|  | Bronchiolitis (PIM2) and surgical or post-operative PICU admission | 32 |
| Surgical or post-operative PICU admission and RSV | RSV AND surgical or post-operative admission | 38 |
| **Total** | | 3,815 |

**eTable 2 -** Patient characteristics and comorbidity in 2,161 children ≤24 months of age with confirmed RSV bronchiolitis

| **Characteristics** | **All (n=2,161)** |
| --- | --- |
| Male | 1,237 (57.2%) |
| Preterm birth | 564 (26.1%) |
| Cardiovascular | |
| Cardiac Disease  *Of which: Hemodynamic significant^a^*  Pre-existent pulmonary hypertension | 276 (12.8%)  *124 (5.7%)*  16 (0.7%) |
| Respiratory | |
| Congenital upper airway disease | 54 (2.5%) |
| Structural lower airway disease | 66 (3.1%) |
| Bronchopulmonary Dysplasia | 41 (1.9%) |
| Pre-existing pulmonary disease | 54 (2.5%) |
| Tracheostomy | 21 (1.0%) |
| Respiratory support at home | 6 (0.3%) |
| Other | |
| Genetic/Syndromal disorder  *Of which: Trisomy 21* | 123 (5.7%)  *36 (1.7%)* |
| Immune compromised | 15 (0.7%) |
| Neurological condition | 99 (4.6%) |
| Pre-existing congenital gastro-intestinal disorder | 11 (0,5%) |

**Legend:** ^a^ Due to the retrospective nature of the study, information on certain cardiac findings was sometimes not complete or difficult to interpret. As such, we additionally collected information of the cases in which the cardiac anomaly was substantial, or when it was documented as hemodynamic significant.

**eTable 3 -** Comparison between different age groups in children admitted to a Dutch PICU for RSV Bronchiolitis 2003-2016from

| **Variable** | **0-3 months**  **(n =1,697)** | **4-12 months**  **(n = 359)** | **13-24 months**  **(n = 105)** | **p** |
| --- | --- | --- | --- | --- |
| LOS^1^, days, median (IQR) | **8 (5-11)** | **7 (4-12)** | **7 (4-13)** | NS |
| **Respiratory support** | **N (%)** | **N (%)** | **N (%)** |  |
| Invasive MV as primary mode of support | 1188 (70) | 209 (58.2) | 52 (49.5) | p <0.01 |
| Invasive MV at any time point during admission | 1265 (74.5) | 229 (63.8) | 57 (54.3) | p <0.01 |
| Non-invasive respiratory support mode as primary mode of support | 299 (17.6) | 80 (22.3) | 30 (28.6) | p <0.01 |
| Re- intubation | 180 (10.6) | 43 (12.0) | 13 (12.4) | NS |
| Re-intubations due to upper airway obstruction | 60 (3.5) | 15 (4.2) | 4 (3.8) | NS |
| **Complications due to invasive MV** |  |  |  |  |
| Upper airway obstruction | 245 (14.4) | 56 (15.6) | 18 (17.1) | NS |
| Non-reversible upper airway obstruction | 26 (1.5) | 2 (0.5) | 2 (1.9) | NS |
| Tracheostomy for subglottic stenosis | 8 (0.5) | 0 (0) | 0 (0) | p =0.02 |
| **Other Complications** |  |  |  |  |
| Mortality | 16 (0.9) | 13 (3.6) | 8 (7.6) | NS |
| ECMO | 18 (1.1) | 10 (2.8) | 4 (3.8) | p <0.01 |
| Resuscitation at PICU ^*^ | 17 (1.0) | 13 (3.6) | 2 (3.6) | p =0.03 |
| Pneumothorax | 23 (1.4) | 12 (3.3) | 2 (1.9) | p =0.03 |
| **Additional use of resources** |  |  |  |  |
| HFO | 144 (8.5) | 41 (11.4) | 7 (6.7) | NS |
| Nitric Oxide | 54 (3.2) | 35 (9.7) | 8 (7.6) | p <0.01 |
| Inotropic medication | 222 (13.1) | 66 (18.4) | 17 (16.2) | P =0.03 |
| Antibiotics  (including before PICU admission) | 1309 (77.1) | 307 (85.5) | 87 (82.9) | P <0.01 |
| Antibiotic treatment started at referring hospital | 495 (29.3) | 137 (38.2) | 49 (46.7) | P <0.01 |
| New antibiotic treatment started at PICU or re-started at PICU | 1005 (59.2) | 236 (65.7) | 63 (34.3) | NS |
| Red blood cell transfusion | 313 (18.4) | 69 (19.2) | 11 (10.5) | NS |
| Platelet transfusion | 20 (1.2) | 11 (3.1) | 4 (3.8) | p < 0.01 |

**Legend**: Pearson Chi Square Tests were carried out to compare groups.. ^1^ Kruskall – Wallis Test instead of Pearson Chi Square Test. % are calculated using the number of children in the specific age group as the denominator. *Resuscitation refers to: cardiac and/or respiratory arrest with subsequent return of spontaneous breathing and/or circulation after medical intervention. NS: not significant.
